# Sudden Cardiac Death Among Firefighters ≤45 Years of Age in the United States

**DOI:** 10.1016/j.amjcard.2013.08.029

**Published:** 2013-09-28

**Authors:** Justin Yang, Dennis Teehan, Andrea Farioli, Dorothee M. Baur, Denise Smith, Stefanos N. Kales

**Affiliations:** a Environmental and Occupational Medicine and Epidemiology Program, Department of Environmental Health, Harvard School of Public Health, Boston, Massachusetts;; b Cambridge Health Alliance, Harvard Medical School, Cambridge, Massachusetts;; c Department of Medical and Surgical Sciences (DIMEC), University of Bologna, Bologna, Italy;; d II. Medizinische Klinik, Klinikum rechts der Isar, Technische Universitaet, Munich, Germany;; e Health and Exercise Sciences Department, Skid-more College, Saratoga Springs, New York.

## Abstract

Sudden cardiac death (SCD) is the leading cause of death in firefighters. Although on-duty SCD usually occurs in older victims almost exclusively because of coronary heart disease, no studies have examined causation across the career span. In the present retrospective case-control study, cases of SCD in young (aged ≤45 years) firefighters from the National Institute for Occupational Safety and Health fatality investigations (n = 87) were compared with 2 age- and gender-matched control groups: occupationally active firefighters (n = 915) and noncardiac traumatic firefighter fatalities (n = 56). Of the SCD cases, 63% were obese and 67% had a coronary heart disease-related cause of death. The SCD victims had much heavier hearts (522 ± 102 g) than noncardiac fatality controls (400 ± 91 g, p <0.001). Cardiomegaly (heart weight >450 g) was found in 66% of the SCD victims and conveyed a fivefold increase (95% confidence interval [CI] 1.93 to 12.4) in SCD risk. Furthermore, hypertension, including cases with left ventricular hypertrophy, increased SCD risk by 12-fold (95% CI 6.23 to 22.3) after multivariate adjustment. A history of cardiovascular disease and smoking were also independently associated with elevated SCD risk (odds ratio 6.89, 95% CI 2.87 to 16.5; and odds ratio 3.53, 95% CI 1.87 to 6.65, respectively). In conclusion, SCD in young firefighters is primarily related to preventable lifestyle factors. Obesity entry standards, smoking bans, and improved screening and/or wellness program are potential strategies to reduce SCD in younger firefighters.

The leading mode of duty-related death among US firefighters is sudden cardiac death (SCD), which accounts for about 50% of on-duty firefighting fatalities.^1–4^ About 90% of these SCD cases will be attributable to coronary heart disease (CHD) and usually occur in firefighters aged >45 years.^4–7^ In addition, emerging evidence has suggested that obesity and left ventricular (LV) hypertrophy and/or cardiomegaly are present in a large proportion of all those with SCD (with and without CHD) in firefighters^8^ and the general population.^9^ Although SCD causation in younger subjects, such as athletes, is usually due to non-CHD structural pathologic features,^10,11^ little is known about SCD in young firefighters. We conducted a case-control study of SCD among firefighters aged ≤45 years to examine the associated cardiovascular disease (CVD) risk factors and underlying pathologic features. Our aims were to (1) describe the specific pathologic-anatomic causes of on-duty SCD in these cases, (2) compare the prevalence and severity of CVD risk factors in SCD fatalities with those in healthy, occupationally active firefighter controls, and (3) compare the cardiac findings from the SCD cases at autopsy with those of firefighters who died of on-duty noncardiac causes.

## Methods

We conducted a retrospective case-control study that serially reviewed and selected as cases all SCD fatalities (aged ≤45 years) from 1996 to 2012 investigated by the National Institute for Occupational Safety and Health (NIOSH).^12^ Two other firefighter groups were chosen as controls: (1) age-matched, career firefighters examined from 2007 to 2009^13^ and (2) age-matched, noncardiac, traumatic fatalities (2004 to 2010) with autopsy reports available.

NIOSH conducts independent investigations of firefighter line-of-duty deaths, and the completed fatality reports are publicly available for download from NIOSH’s Firefighter Fatality Investigation and Prevention Program website.^12^ Two physician investigators (J.Y. and D.T.) examined in detail all fatality reports published online from January 1996 to December 2012 to determine whether each case met our inclusion criteria as listed in the following paragraph. A third physician investigator (A.F.) then reviewed the NIOSH database and previously selected cases again, with final decisions on inclusion resolved by the senior investigator (S.N.K.). SCD case data were extracted using a standardized electronic template^5,8^ by 2 of us (D.T. and J.Y.) independently and then were verified for completeness and accuracy (by J.Y.). Any disagreements among the investigators on data extraction were resolved by the senior physician investigator (S.N.K.).

The inclusion criteria for the NIOSH SCD fatality cases were (1) NIOSH investigated cases published on the website from January 1996 to December 2012, (2) firefighters who experienced SCD and died within 24 hours of their last fire service duty or experienced a sudden cardiac event within 24 hours of their last duty and the event was associated with loss of consciousness within 1 hour of onset, and, subsequently, the firefighter never regained consciousness before biologic death, (3) age ≤45 years, and (4) autopsy report or sufficient medical findings available to determine the underlying cause of death.

An existing database previously assembled from career fire departments was reviewed for occupationally active control firefighters. The cohort’s cardiovascular and health status were comprehensively characterized by baseline fire department medical examinations.^13,14^ The inclusion criteria for the occupationally active firefighter controls were (1) age ≤45 years, and (2) no medical restrictions or physical limitations on duty.

Potential age-matched, noncardiac traumatic fatalities (deaths due to blunt trauma, burns, or asphyxiation) were identified for 2004 to 2010 from a firefighter autopsy research data bank maintained by 1 of us (D.S.) and the National Fallen Firefighters Foundation. The inclusion criteria for the National Fallen Firefighters Foundation noncardiac traumatic controls were (1) age ≤45 years, (2) death while on duty, and (3) cause of death determined by autopsy to be due to blunt trauma, burns, or asphyxiation and not related to any cardiovascular pathologic entity.

Among the occupationally active controls, firefighters were considered active smokers if they self-reported smoking within the previous 12 months. Diabetes mellitus was defined using the Framingham criteria (random blood glucose ≥150 mg/dl, previous diagnosis of diabetes, and/or requiring diabetes mellitus medications).^5,8^ Hypertension was considered present if firefighters had a systolic blood pressure of ≥140 mm Hg and/or diastolic blood pressure of ≥90 mm Hg at rest, a previous hypertension diagnosis, and/or required hypertension medication. Firefighters with a total cholesterol level of ≥200 mg/dl, low-density lipoprotein of ≥160 mg/dl, a previous diagnosis of hyperlipidemia, and/or requiring lipid-lowering medications were considered to have dyslipidemia.

In the NIOSH SCD cases, the determinations were according to the same criteria or a description of the risk factors as presented by the NIOSH investigators anywhere in the case report. We also considered a second definition of hypertension that included those with hypertension as defined plus those with LV hypertrophy found on autopsy.^8^ Any firefighter was considered to have a history of CHD or CHD equivalent if the NIOSH report or medical record reported previous myocardial infarction, angioplasty, stent placement, or a clinical diagnosis of CHD on the basis of an abnormal calcium score or exercise tolerance test findings. A history of valvular disease was considered present if a previous diagnosis of valvular abnormalities and/or disease or appropriate autopsy findings of valvular disease were present. A “history of chest pain or shortness of breath” was considered present if the firefighter had had episodes of chest pain and/or shortness of breath documented without a CHD diagnosis. We conservatively coded the CVD risk factors in the SCD cases as negative when these were undeterminable or ambiguous from the investigation report.

The use of de-identified data from the occupationally active firefighter controls was previously approved by the institutional review board of Harvard School of Public Health and local institutional review boards, as appropriate. The investigations and autopsy reports from NIOSH and the National Fallen Firefighters Foundation were exempt from institutional review board review (deceased, nonliving subjects).^15^

Statistical analyses were performed using SPSS, version 21.0 (IBM, Armonk, New York) and Stata, version 12.1 SE (StataCorp, College Station, Texas). Categorical variables were compared using Fisher’s exact test and normally distributed continuous variables using Student’s *t* test. Associations of risk factors with SCD were characterized by odds ratios and associated 95% confidence intervals. Variables to be introduced in the multivariate logistic regression models were selected a priori. p Values <0.05 were considered statistically significant, and all statistical tests were 2-sided.

## Results

A total of 87 SCD fatality cases, 915 occupationally active controls, and 56 trauma deaths met the inclusion criteria. The SCD cases dichotomized by age are listed in Table 1. Cases with cardiomyopathy and/or cardiomegaly in the absence of CHD were most often associated with hypertrophic cardiomyopathy or nonspecific cardiomyopathies. Overall, 67% of SCD cases had CHD as a contributing factor (categories 1 and 4).

The CVD risk factor prevalence between the NIOSH SCD cases and occupationally active firefighter controls is presented in Table 2. The odds ratios for obesity, smoking, and hypertension were all statistically significant for an association with SCD after multivariate adjustment. A history of CHD, CHD equivalent, or valvular disease diagnosed before death was associated with a sevenfold increase in the risk of SCD, even after multivariate adjustment (95% confidence interval 2.87 to 16.5).

Data from the SCD cases and noncardiac trauma controls are listed in Table 3. Our SCD cases had significantly larger hearts (mean difference >120 g) compared with the trauma controls (p <0.001). Furthermore, the odds of SCD increased almost fivefold in the presence of cardiomegaly (heart weight >450 g) after multivariate adjustment (95% confidence interval 1.93 to 12.4). Finally, in both groups we observed cardiomegaly primarily in obese victims: 74% of cases and 67% of controls with an enlarged heart were obese.

## Discussion

The results from the present study support the finding that on-duty SCD in younger US firefighters, even those aged <35 years, is primarily related to preventable lifestyle factors, which culminate in obesity, CHD, and LV hypertrophy and/or cardiomegaly. Our study also identified several important risk factors for SCD. Those with SCD were more likely to be obese, hypertensive, and smokers and to have a history of significant CVD than were active controls. Furthermore, when the SCD autopsy results were compared with those from other firefighters who died of noncardiac causes, the SCD cases were more obese, had significantly greater heart weights, and had an increased risk of cardiomegaly (heart weight >450 g). Two thirds of the SCD cases had a heart weight >450 g and >40% weighed >550 g.

In strong agreement with data from the general population,^9^ we found that the most common underlying pathologic reason for SCD in younger firefighters was CHD, together with cardiomegaly (53%). Most remaining SCD cases were due to either CHD or some type of cardiomegaly alone. These results for cardiomegaly and obesity were also consistent with an earlier study of SCD in the fire service that was limited to CHD deaths, in which the average age of those dying was 50 years and >75% of the subjects were >45 years old.^8^

In our present study, 63% of those with SCD were obese compared with 36% of the active firefighter controls. This yielded a twofold increased risk of SCD after adjustment for covariates. Also, 28% of the SCD cases had class II or III obesity compared with only 10% of our occupationally active control firefighters. Our results can most likely be explained by obesity’s well-known associations with the clustering of cardiometabolic risk factors, especially hypertension and obstructive sleep apnea, which increase the risk of both CHD and cardiomegaly, as well as death.^16,17^

Accordingly, LV hypertrophy was another powerful predictor of SCD, in agreement with previous studies.^3,18–20^ In previous investigations of CHD fatalities among firefighters, LV hypertrophy was found in 60% to 76% of autopsies.^3,5,8^ In our study, 70% of cases had evidence of LV hypertrophy. The autopsy findings from our trauma controls (22% prevalence of heart weight >450 g) indicated that LV hypertrophy and/or cardiomegaly could be fairly common among firefighters aged ≤45 years. Additional research is needed to develop more sensitive screening methods for cardiomegaly in the fire service.

Our observation of high body mass indexes, heavier heart weights, and a fivefold risk increase associated with cardiomegaly in SCD cases, although already significant, were actually likely to be conservative. This resulted from the greater obesity we observed in the traumatic controls compared with the occupationally active cohort. Because it is not possible to obtain the heart weight from the active controls, an excess of extreme obesity in noncardiac fatalities potentially biased our findings toward the null hypothesis, because obesity usually increases the heart weight. We hypothesized that traumatic fatalities occur more frequently in obese firefighters because they are more inclined to be physically trapped during a fire secondary to their body size and relative physical immobility.^21^

Our study had several minor limitations. First, the NIOSH investigation program might have underrepresented volunteer firefighters. Only about 23% of volunteer firefighter deaths were examined by NIOSH from 2004 to 2009 compared with 55% of career fatalities.^22^ However, although volunteers account for most (about 70%) of the United States fire service,^23^ we also know that the volunteers were older as a group and 93% of the SCD victims who were aged >60 years were volunteers.^4^ These statistics suggest a skewed distribution of SCD fatalities toward older volunteers, whose cases the NIOSH is less likely to review. Therefore, any selection bias would have been limited to the NIOSH investigations of younger firefighters.

Another limitation of our study was that we were only able to extract the autopsy results provided in the NIOSH investigations, and no standardized reporting formats were used. Thus, it is possible that our results underestimated the true magnitude of obesity or cardiomegaly in young firefighters. Regardless, our significant findings, combined with the results from previous studies, strongly suggest that obesity and cardiomegaly increase the odds of SCD.^3,5,16,24,25^

Our study also had major methodologic strengths. We created a statistically rigorous case-control design with 2 age-matched control groups from the same occupation whose medical data were derived from similar periods. Also, although we conservatively coded the incomplete reports from the NIOSH as negative, we adopted broad definitions for the risk factors in the active control group. Our near complete risk factor data from the controls would have most likely biased our results toward the null hypothesis.

The present results continue to support previous recommendations^1,3,12,16,22,26–29^ for mandating medical screening and wellness programs for firefighters, because these remain relatively uncommon within the United States fire service.^1,22^ In particular, imposing an entry-level obesity standard should be strongly supported because of (1) the prevalence of obesity reported in the present study and in previous studies,^2,16,28^ and (2) our present findings that obesity is strongly associated with an increased risk of cardiomegaly and SCD.

## Figures and Tables

**Table 1:** Descriptive results of sudden cardiac death (SCD) cases aged ≤45 years from the National Institute for Occupational Safety and Health (NIOSH) investigations

Variable	Age <35 yrs (n = 22)	Age 35–45 yrs (n = 65)	Total (n = 87)
Age (yrs)	28.0 ± 4.1	41.0 ± 3.1	37.7 ± 6.6
Men (%)	21 (96)	64 (99)	85 (98)
BMI (kg/m^2^)	31.6 ± 6.5 (n = 18)	32.1 ± 6.1 (n = 50)	32.0 ± 6.2 (n = 68)
Obesity category			
BMI ≥30 kg/m^2^ (obese)	12 (67)	31 (62)	43 (63)
BMI ≥30 but <35 kg/m^2^	7 (39)	17 (34)	24 (35)
BMI ≥35 but <40 kg/m^2^	3 (17)	7 (14)	10 (15)
BMI ≥40 kg/m^2^	2 (11)	7 (14)	9 (13)
Smoker	4 (18)	20 (31)	24 (28)
Career status			
Career	11 (50)	41 (63)	52 (60)
Volunteer	11 (50)	24 (37)	35 (40)
Autopsy findings			
CHD only	1 (5)	11 (17)	12 (14)
Hypertrophic cardiomyopathy	3 (14)	2 (3)	5 (6)
LV hypertrophy or hypertension heart disease	1 (5)	3 (5)	4 (5)
CHD + cardiomegaly	7 (32)	39 (60)	46 (53)
Arrhythmia because of idiopathic dilated cardiomyopathy	3 (14)	1 (2)	4 (5)
Coronary anomaly	1 (5)	1 (2)	2 (2)
Valvular disease (acquired)	3 (14)	3 (5)	6 (7)
Congenital heart disease other than coronary anomaly	0	1 (2)	1 (1)
WPW, long QT, and other primary arrhythmia without structural disease	1 (5)	0	1 (1)
Cardiac sarcoidosis	0	2 (3)	2 (2)
Commotio cordis	0	0	0
Myocarditis	0	0	0
Other cardiovascular causes	1 (5)	1 (2)	2 (2)
Cause of SCD not confirmed*	1 (5)	1 (2)	2 (2)
Heart weight (g)	490 ± 84 (n = 18)	534 ± 107 (n = 47)	522 ± 102 (n = 65)
Heart weight group			
>450 g	11 (61)	32 (68)	43 (66)
>550 g	5 (28)	22 (47)	27 (42)

Data are presented as mean ± SD or n (%).

BMI = body mass index; WPW = Wolff-Parkinson-White syndrome.

* Cause of SCD could not be confirmed because of lack of autopsy and previous medical records.

**Table 2:** Statistical analyses of cardiovascular disease (CVD) risk factors comparing sudden cardiac death (SCD) cases aged ≤45 years from National Institute for Occupational Safety and Health (NIOSH) investigations with occupationally active firefighter controls

Variable	NIOSH Cases (n = 87)	Active Controls (n = 915)	Univariate Analysis			Multivariate Analysis					
						Model I*^,†^			Model II^†,‡^		
			OR	95% CI	p	OR	95% CI	p	OR	95% CI	p
Risk factor											
Age (yrs)	37.7 ± 6.6	35.4 ± 6.1	1.06	1.03–1.11	0.001	1.03	0.98–1.08	0.268	1.02	0.97–1.07	0.451
Men	85/87 (98)	886/915 (97)	0.72	0.17–3.06	0.655	—	—	—	—	—	—
BMI (kg/m^2^)											
<30	25/68 (37)	582/908 (64)	1.00	Ref		1.00	Ref		1.00	Ref	
≥30	43/68 (63)	326/908 (36)	3.07	1.84–5.12	<0.001	2.20	1.27–3.81	0.005	1.76	0.99–3.11	0.053
Smoker^§^	24/87 (28)	70/779 (9)	3.86	2.27–6.56	<0.001	3.53	1.87–6.65	<0.001	3.50	1.76–6.95	<0.001
Diabetes mellitus^‖^	4/87 (5)	18/908 (2)	2.38	0.79–7.21	0.124	3.26	0.93–11.5	0.066	2.17	0.59–7.95	0.243
Hypertension^¶^	41/87 (48)	179/904 (20)	3.69	2.34–5.81	<0.001	3.43	2.01–5.87	<0.001	—	—	—
Hypertension 2^#^	62/87 (72)	179/904 (20)	10.5	6.35–17.2	<0.001	—	—	—	11.8	6.23–22.3	<0.001
Dyslipidemia**	46/87 (53)	366/908 (40)	1.66	1.07–2.58	0.024	1.47	0.86–2.51	0.157	1.53	0.88–2.68	0.134
History of CVD											
CHD, CHD equivalent, or valvular disease^††^	18/87 (21)	29/786 (4)	6.81	3.60–12.9	<0.001	6.89	2.87–16.5	<0.001	5.72	2.40–13.6	<0.001
Irregular rhythm	2/87 (2)	45/786 (6)	0.39	0.09–1.63	0.195	0.13	0.02–1.06	0.057	0.13	0.02–1.04	0.054
Abnormal findings on ECG or echocardiogram	6/87 (7)	62/786 (8)	0.86	0.36–2.06	0.744	0.50	0.16–1.59	0.242	0.41	0.13–1.31	0.132
Chest pain or shortness of breath^‡‡^	7/87 (8)	9/786 (1)	7.55	2.74–20.8	<0.001	1.92	0.46–8.01	0.372	2.15	0.50–9.29	0.307

Data are presented as mean ± SD or n (%), unless otherwise noted.

CI = confidence interval; ECG = electrocardiogram; hypertension 2 = second definition of hypertension; OR = odds ratio; Ref = reference category.

* Adjusted by age, BMI (dichotomous), smoking, diabetes mellitus, hypertension, and dyslipidemia.

† Analysis restricted to 68 cases and 765 controls with complete information.

‡ Adjusted by age, BMI (dichotomous), smoking, diabetes mellitus, hypertension 2, and dyslipidemia.

§ Subject was smoking within previous 12 months.

‖ Cases: evidence of diabetes mellitus in report; controls: blood glucose ≥150 mg/dl, previous diabetes mellitus diagnosis, and/or taking medication.

¶ Systolic blood pressure ≥140 mm Hg and/or diastolic blood pressure ≥90 mm Hg, previous hypertension diagnosis, and/or taking medication.

# Included all cases of hypertension defined in previous footnote plus those with findings of LV hypertrophy on autopsy.

** Evidence of dyslipidemia mentioned (total cholesterol ≥200 mg/dl or low-density lipoprotein ≥160 mg/dl), previous diagnosis of hyperlipidemia, and/or taking medication.

†† CHD, CHD equivalent: previous myocardial infarction, angioplasty, stent placement, or clinical diagnosis of CHD because of abnormal calcium score or exercise tolerance test findings; valvular disease: previous diagnosis of valvular abnormalities or disease or presence of appropriate autopsy findings.

‡‡ Episodes of chest pain or shortness of breath documented but without a CHD diagnosis.

**Table 3:** Statistical analyses comparing sudden cardiac death (SCD) cases aged ≤45 years from National Institute for Occupational Safety and Health (NIOSH) investigations and traumatic fatality controls

Variable	NIOSH SCD Cases (n = 87)	Trauma Controls (n = 56)	Univariate Analysis			Multivariate Analysis		
			OR	95% CI	p	OR	95% CI	p
Age (yrs)	37.7 ± 6.6	31.2 ± 8.3	1.12	1.07–1.17	<0.001	1.09*	1.03–1.15*	0.002
BMI (kg/m^2^)								
<30	25/68 (37)	33/56 (59)	1.00	Ref		1.00	Ref	
≥30	43/68 (63)	23/56 (41)	2.47	1.19–5.10	0.015	1.23*	0.50–3.07*	0.650
Heart weight (g)	522 ± 102	400 ± 91	1.01	1.01–1.02	<0.001	1.01^†^	1.01–1.02^†^	<0.001
Heart weight group								
≤450	22/65 (34)	42/54 (78)	1.00	Ref		1.00	Ref	
>450	43/65 (66)	12/54 (22)	6.84	3.01–15.6	<0.001	4.89*	1.93–12.4*	0.001

Data are presented as mean ± SD or n (%).

CI = confidence interval; OR = odds ratio; Ref = reference category.

* Estimates from logistic regression model that included age, BMI, and dichotomized heart weight; analysis restricted to 59 cases and 54 controls with complete information.

† Estimates from logistic regression model that included age, BMI, and heart weight; analysis restricted to 59 cases and 54 controls with complete information.
